# Commercial 4-dimensional echocardiography for murine heart volumetric evaluation after myocardial infarction

**DOI:** 10.1186/s12947-020-00191-5

**Published:** 2020-03-12

**Authors:** Cody Rutledge, George Cater, Brenda McMahon, Lanping Guo, Seyed Mehdi Nouraie, Yijen Wu, Flordeliza Villanueva, Brett A. Kaufman

**Affiliations:** 1 Vascular Medicine Institute, University of Pittsburgh, Pittsburgh, PA USA; 2 Division of Cardiology, Cardiovascular Institute, University of Pittsburgh, Pittsburgh, PA USA; 3 Division of Pulmonary, Allergy, and Critical Care Medicine, University of Pittsburgh, Pittsburgh, PA USA; 4 Department of Developmental Biology, University of Pittsburgh, Pittsburgh, PA USA

**Keywords:** 4-dimensional ultrasound, Preclinical echocardiography, Mouse coronary artery ligation

## Abstract

**Background:**

Traditional preclinical echocardiography (ECHO) modalities, including 1-dimensional motion-mode (M-Mode) and 2-dimensional long axis (2D-US), rely on geometric and temporal assumptions about the heart for volumetric measurements. Surgical animal models, such as the mouse coronary artery ligation (CAL) model of myocardial infarction, result in morphologic changes that do not fit these geometric assumptions. New ECHO technology, including 4-dimensional ultrasound (4D-US), improves on these traditional models. This paper aims to compare commercially available 4D-US to M-mode and 2D-US in a mouse model of CAL.

**Methods:**

37 mice underwent CAL surgery, of which 32 survived to a 4 week post-operative time point. ECHO was completed at baseline, 1 week, and 4 weeks after CAL. M-mode, 2D-US, and 4D-US were taken at each time point and evaluated by two separate echocardiographers. At 4 weeks, a subset (*n* = 12) of mice underwent cardiac magnetic resonance (CMR) imaging to serve as a reference standard. End systolic volume (ESV), end diastolic volume (EDV), and ejection fraction (EF) were compared among imaging modalities. Hearts were also collected for histologic evaluation of scar size (*n* = 16) and compared to ECHO-derived wall motion severity index (WMSI) and global longitudinal strain as well as gadolinium-enhanced CMR to compare scar assessment modalities.

**Results:**

4D-US provides close agreement of ESV (Bias: -2.55%, LOA: − 61.55 to 66.66) and EF (US Bias: 11.23%, LOA − 43.10 to 102.8) 4 weeks after CAL when compared to CMR, outperforming 2D-US and M-mode estimations. 4D-US has lower inter-user variability as measured by intraclass correlation (ICC) in the evaluation of EDV (0.91) and ESV (0.93) when compared to other modalities. 4D-US also allows for rapid assessment of WMSI, which correlates strongly with infarct size by histology (r = 0.77).

**Conclusion:**

4D-US outperforms M-Mode and 2D-US for volumetric analysis 4 weeks after CAL and has higher inter-user reliability. 4D-US allows for rapid calculation of WMSI, which correlates well with histologic scar size.

## Background

Echocardiography (ECHO) is a highly efficient and reliable tool for clinical and pre-clinical evaluation of cardiac function [1–3]. Advances in high-frequency and high-resolution image acquisition continue to increase temporal and spatial resolution in small rodent models [1, 4, 5]. However, traditional ECHO modalities for evaluating ventricular size and function, including 1-dimensional motion-mode (M-mode) and 2-dimensional (2D-US) long-axis analysis, make geometric assumptions about the heart that limit their accuracy, particularly in the setting of heart disease, where regional ventricular shape abnormalities may exist [6–8]. For example, mouse models of cardiac pathology, particularly myocardial infarction (MI), frequently result in abnormal ventricular remodeling that deviates from the geometric assumption that the left ventricle has an ellipsoid shape, limiting the utility of conventional echocardiography in such settings and requiring careful consideration of modality choice [9, 10].

Three-dimensional (3D) ECHO has gained favor recently in clinical and pre-clinical models, allowing full volume visualization of the heart by stacking concentric short-axis images to form a 3D representation at static time points [4, 11, 12]. 3D ECHO has been further improved by combining respiratory- and ECG-gated image acquisition to create 3D images throughout the cardiac cycle, referred to as 4-dimensional ultrasound (4D-US) [13, 14]. 4D-US has recently became commercially available for pre-clinical models and has already been validated against cardiac magnetic resonance (CMR) imaging in wild type mice and a mouse model of hypertrophy [15]. While CMR imaging remains the gold standard for assessment of cardiac function in mice [16–18], recent studies in 3D ECHO and 4D-US have shown considerable advances in scanning time and reliability [13–15].

In this study, we aimed to validate commercially available 4D-US in a mouse model of myocardial infarction. We performed coronary artery ligation (CAL) surgery on mice to evaluate traditional M-mode, 2D-US, and 4D-US using the Vevo 3100 preclinical imaging system against CMR imaging to compare the assessment of volumetric parameters at 4 weeks after CAL.

## Methods

### Coronary artery ligation and mouse model

All animal use was performed at the University of Pittsburgh in compliance with the National Institutes of Health Guide for Care and Use of Experimental Animals and was approved by the University of Pittsburgh Animal Care and Use Committee. Previously characterized mice harboring a floxed TFAM gene were crossed with a-myosin heavy chain Cre transgene (MHC-Cre) to generate mice that were MHC-Cre (+) x Flox-TFAM (*n* = 22) and MHC-Cre (−) x Flox-TFAM (*n* = 15). Both males and females were used in this study. Mice were aged to twelve weeks, at which point they underwent baseline echocardiography followed by coronary artery ligation surgery. Mice were anesthetized and ventilated prior to open thoracotomy through the 4th rib followed by opening of the pericardium and coronary artery ligation (CAL) by suture placement around the proximal coronary artery [19]. All surviving mice underwent follow-up echocardiography at 1 and 4 weeks.

### Echocardiography

Mice were anesthetized using isoflurane delivered by nose cone at the following timepoints: baseline (*n* = 37), 1 week following CAL (*n* = 32), and 4 weeks following CAL (*n* = 32). At each timepoint, depilatory cream was applied to the thorax to remove hair. Animals were maintained at 37 °C via heating pad and rectal probe and were monitored using surface ECG limb electrodes throughout imaging. Transthoracic echocardiography was performed using the Vevo 3100 imaging systems (FUJIFILM VisualSonics, Toronto, Canada) with a probe attached to a step motor. The Visualsonic MX400 (20–46 MHz, 50 μm axial resolution) linear array transducer was used for all image acquisition. Heart rate was maintained between 400 and 500 bpm during imaging by adjusting isoflurane concentration to a final concentration of 1–2%. M-mode and B-mode images of the heart were obtained for at least ten cardiac cycles in the parasternal long axis and mid-papillary muscle level short axis views. For 4D image acquisition, the step motor was positioned just below the apex and the motor aligned to take concentric short axis images in 0.2 mm steps. At each position, a complete cardiac cycle was recorded using automated ECG and respiratory gating. 4D images were constructed using Vevo 4D image software. Image analysis was performed independently by two blinded sonographers (CR and BM). End-diastolic volume (EDV), End-systolic volume (ESV), and Ejection Fraction (EF), were calculated using the following formulas:

M-Mode (short-axis):

$$ \mathsf{EDV}=\left[\ \mathsf{7.0}/\left(\mathsf{2.4}+\mathsf{LVID};\mathsf{d}\right)\ \mathsf{x}\ \mathsf{LVID};{\mathsf{d}}^{\mathsf{3}}\right]\ \mathsf{where}\ \mathsf{LVID};\mathsf{d}=\mathsf{Left}\ \mathsf{ventricular}\ \mathsf{internal}\ \mathsf{diameter}\ \mathsf{at}\ \mathsf{end}\ \mathsf{diastole} $$

$$ \mathsf{ESV}=\left[\ \mathsf{7.0}/\left(\mathsf{2.4}+\mathsf{LVID};\mathsf{s}\right)\ \mathsf{x}\ \mathsf{LVID};{\mathsf{s}}^{\mathsf{3}}\right]\ \mathsf{where}\ \mathsf{LVID};\mathsf{s}=\mathsf{Left}\ \mathsf{ventricular}\ \mathsf{internal}\ \mathsf{diameter}\ \mathsf{at}\ \mathsf{end}\ \mathsf{systole} $$

$$ \mathsf{EF}=\mathsf{100}\ \mathsf{x}\ \left(\left(\mathsf{EDV}-\mathsf{ESV}\right)/\mathsf{EDV}\right) $$

2D-US analysis of the parasternal long-axis was completed using operator-defined LV trace function of Vevo LAB software (v3.2.0) and calculations of EDV, ESV, and EF made by Simpson’s method. 4D-US measurements were calculated directly from volumetric measurements based on operator-defined edge-tracing using Vevo 4D imaging software.

The Vevo Strain software was used to measure both longitudinal and radial strain by using long-axis images and semi-automated border tracking. The LV was visualized during end-diastole and the endocardial and epicardial borders were determined. A minimum of five cardiac cycles were then traced automatically by the speckle-tracking software and reviewed by the user. Respiratory variation was excluded from these cycles. Global peak longitudinal and radial strain were calculated using Vevo Strain software on images taken from the long-axis. Global peak circumferential and radial strain were calculated from images from the short axis. Further, the LV was automatically divided into six segments in the long-axis images by the software. The standard deviation of the strain among the individual segments was calculated as a measure of LV dyssynchrony.

### WMSI

WMSI was calculated using a 16-segment model collected from 3 short axis views collected during 4D-US image acquisition (*n* = 32). Short axis views were obtained 1 mm, 3 mm, and 5 mm from the apex for standardization. The most distal image was divided into 4 sections and the remaining sections divided into 6 sections as previously described (Fig. 3) [20, 21]. Individual sections were graded as: 1- normal, 2- hypokinetic, 3- akinetic, 4- dyskinetic, 5- aneurysmal. WMSI was calculated as average of all 16-segment motion scores.

### CMR

Mice (*n* = 12) were anesthetized with 4% isoflurane mixed with room air in an induction box for 1 to 3 min. The depth of anesthesia was monitored by toe reflex, extension of limbs, and spine positioning. Anesthesia was maintained by 1.5 to 2% isoflurane and 100% oxygen via a nose cone. Respiration waveforms were continuously monitored using a small pneumatic pillow under the animal’s diaphragm connected to a magnet-compatible pressure transducer (SA Instruments, Stony Brook, NY). CMR was performed on a Bruker Biospec 7 T/30 system (Bruker Biospin MRI, Billerica, MA) with a 35-mm quadrature coil for both transmission and reception. The Bruker Intragate module was used for image-gated cine MRI with retrospective navigation. Subcutaneous injection of Multi-Hance (Gadobenate dimeglumine, 529 mg/ml, Bracco Diagnostics, Inc., Monroe Twp, NJ 08831) was administered immediately before the CMR acquisition at 0.1 mmol Gd/kg bodyweight. T_1_-weighted images to highlight LGE were acquired 15–20 min after the subcutaneous administration of Multi-Hance. Eight T_1_-weighted short-axis imaging planes covering the whole ventricular volume with no gaps were acquired with the following parameters: Field of view (FOV) = 2.5 cm X 2.5 cm, slice thickness = 1 mm, in-plane resolution = 0.97 μm, flip angle (FA) = 10 degrees, echo time (TE) = 3.059 msec, repetition time (TR) = 5.653 msec. White-blood cine movies with 20 cardiac phases were acquired for each mouse with equivalent temporal resolution for the cine loops was about 16.5–21.5 ms per frame. Eight short-axis imaging planes covering the whole ventricular volume with no gaps and one long-axis plane were acquired with the following parameters: Field of view (FOV) = 2.5 cm X 2.5 cm, slice thickness = 1 mm, in-plane resolution = 0.97 μm, flip angle (FA) = 30 degrees, echo time (TE) = 1.872 msec, repetition time (TR) = 38.293 msec.

The extent of myocardial infarction was defined by the percentage of the myocardium displaying hyperintensity 15–20 min after Gd administration. To obtain the proportion of myocardial infarction, the area of hyperintensity was manually traced by a blinded operator on the Paravision 5.1 Xtip software (Bruker Biospin MRI, Billerica, MA). The extent of myocardial hemorrhage was defined by dark hypointensity on the cine images. To obtain the proportion of myocardial hemorrhage, the area of hypointensity was manually traced by a blinded operator on the software. The left ventricular endocardium and epicardium boundaries of each imaging slice at the end-systole (ES) and the end-diastole (ED) were manually traced by a blinded operator in the software to calculate the following functional parameters: left ventricular blood volume (LVV), left ventricular wall volume (LV wall), LV mass, stroke volume (SV), ejection fraction, heart rate (HR), cardiac output (CO), longitudinal shortening, and radial shortening. LVV is calculated by summation of all the short-axis slices. The EF was calculated using the following equation: $EF=\frac{\sum \limits_i{A}_i^{es}{h}_i}{\sum \limits_i{A}_i^{ed}{h}_i}\times 100\%$, where ${A}_i^{es}$ is the internal left ventricle area of slice *i* at end systole, ${A}_i^{ed}$ the internal left ventricle area of slice *i* at end diastole, and *h*_*i*_ is the thickness of each scanned slice.

### Tissue histology

LV tissue (*n* = 16) was fixed overnight in 10% formaldehyde at 4 °C. Tissues were then washed with PBS and transferred to 70% EtOH and stored at room temperature. After fixation, tissues were brought to the Department of Pathology Histology Core at the University of Pittsburgh and sectioned into 10 μm slices at 1 mm intervals throughout the myocardium. Sections were stained with hematoxylin and eosin (H&E) or Masson’s Trichrome stains and images obtained on a TissueFAXS Histo (TissueGnostics, Vienna, Austria) upright brightfield microscope utilizing HistoQuest software. Image analysis was performed by automated red and blue channel separation using Image Measurement 9.0 (Bersoft Imaging, Cologne, Germany).

### Statistical analysis

Differences between imaging modalities were evaluated by Bland-Altman analysis and are expressed as % bias and 95% level of agreement (LOA). Bland-Altman percentage bias was calculated as (100*(B-A)/Average vs Average) where “B” represents measurements from 4D-US, 2D-US, or M-mode, and “A” represents measurements from CMR. Intraclass correlation was used to evaluate reliability between single measurements of EDV, ESV, and EF between two users (CR and BM). Normality was assessed using D’Agostino-Pearson omnibus K2 testing for each data set. Regression analysis was performed using Spearman rank correlation to compare scar size among several methods (Fig. 4, Supplemental Table 2). Correlation was graded as poor (0.0–0.5), moderate (0.5–0.7), strong (0.7 to 0.9), or very strong (0.9–1.0). Supplemental Fig. 2 data are expressed and mean ± standard error. Power analysis was completed based on linear regression analysis to confirm group size (two-sided test, α = 0.05, based on values for EDV). For all statistical tests, *p* ≤ 0.05 was considered significant. All normality, regression and statistical tests were completed using Graphpad Prism 7 software (San Diego, CA) except for ICC, which was calculated using Microsoft Excel (Redmond, WA), and power analysis which was calculated using StataCorp Stata 16.0 (College Station, Texas).

## Results

### Animal model of myocardial infarction

32 of the initial 37 mice survived to 4 weeks post-CAL (86.4%, Table 1). The mouse background was MHC-CRE (+) x Flox-TFAM or MHC-CRE (−) x Flox-TFAM. Subgroup analysis was performed between MHC (+) and MHC (−) groups and there was no significant difference noted in survival, scar size, baseline EDV, ESV, or EF, as well as 4-week EDV, ESV, or EF between groups by any ECHO modality (Supplemental Fig. 2, Supplemental Table 1).

**Table 1:** Baseline and 4-week post CAL Animal Data

	Total Mice	Percent
Survival	32/37	86.40%
	**Mean**	**SEM**
Baseline Weight (g)	25.26	0.58
Week 4 Weight (g)	25.44	0.54
Baseline HR (bpm)	454.96	7.96
Week 4 HR; M-Mode	451.73	8.35
Week 4 HR; 2D-US	459.73	9.19
Week 4 HR; 4D-US	459.53	6.28
Week 4 HR; CMR	356.21	8.20
	**Mean** ± **SEM**	**Range**
Histologic Scar Size	21.54 ± 4.07%	4.02–57.0%
WMSI	1.32 ± 0.05	1.065–2.0625
CMR Hyperintense Volume	13.53 ± 2.22%	5.52–28.10%
Global Peak Longitudinal Strain	−5.27 ± 1.10%	−13.29% – -1.072%

### Comparison of ECHO modalities to CMR

**Fig. 1:**
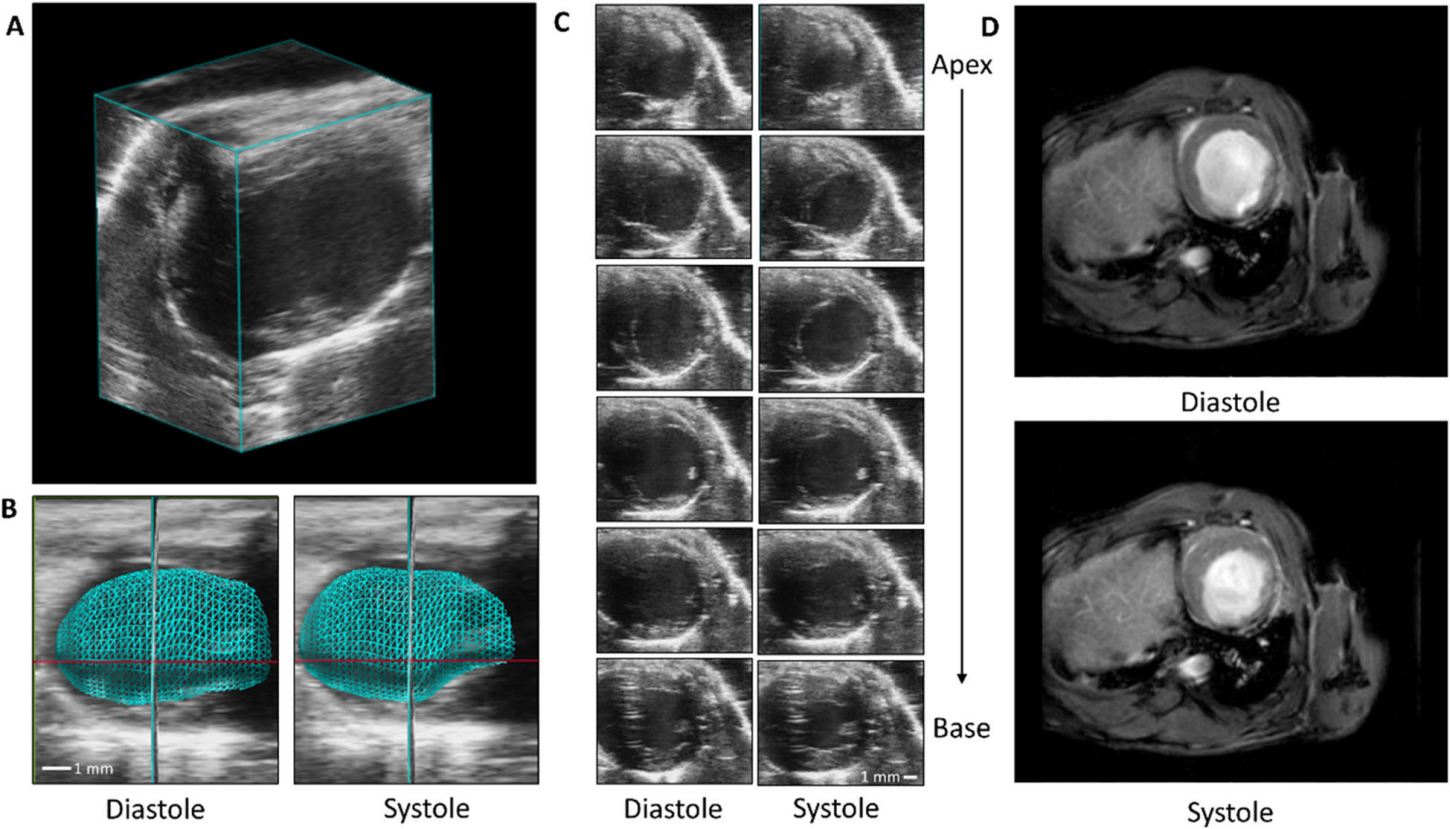
Representive Images Using 4D-US and CMR. **a** 4D-US cube reconstruction of concentric stacked images in an infarcted heart. **b** Representative wire tracing of 4D-US reconstruction in an infarcted heart 4 weeks after CAL. **c** Representative short-axis images taken during end-diastole (left column) and end-systole (right column) at 1 mm intervals from the apex (top) to base (bottom) of an infarcted heart 4 weeks after CAL. **d** Representative mid-ventricular CMR images of an infarcted heart during end-diastole (top) and end-systole (bottom)

**Fig. 2:**
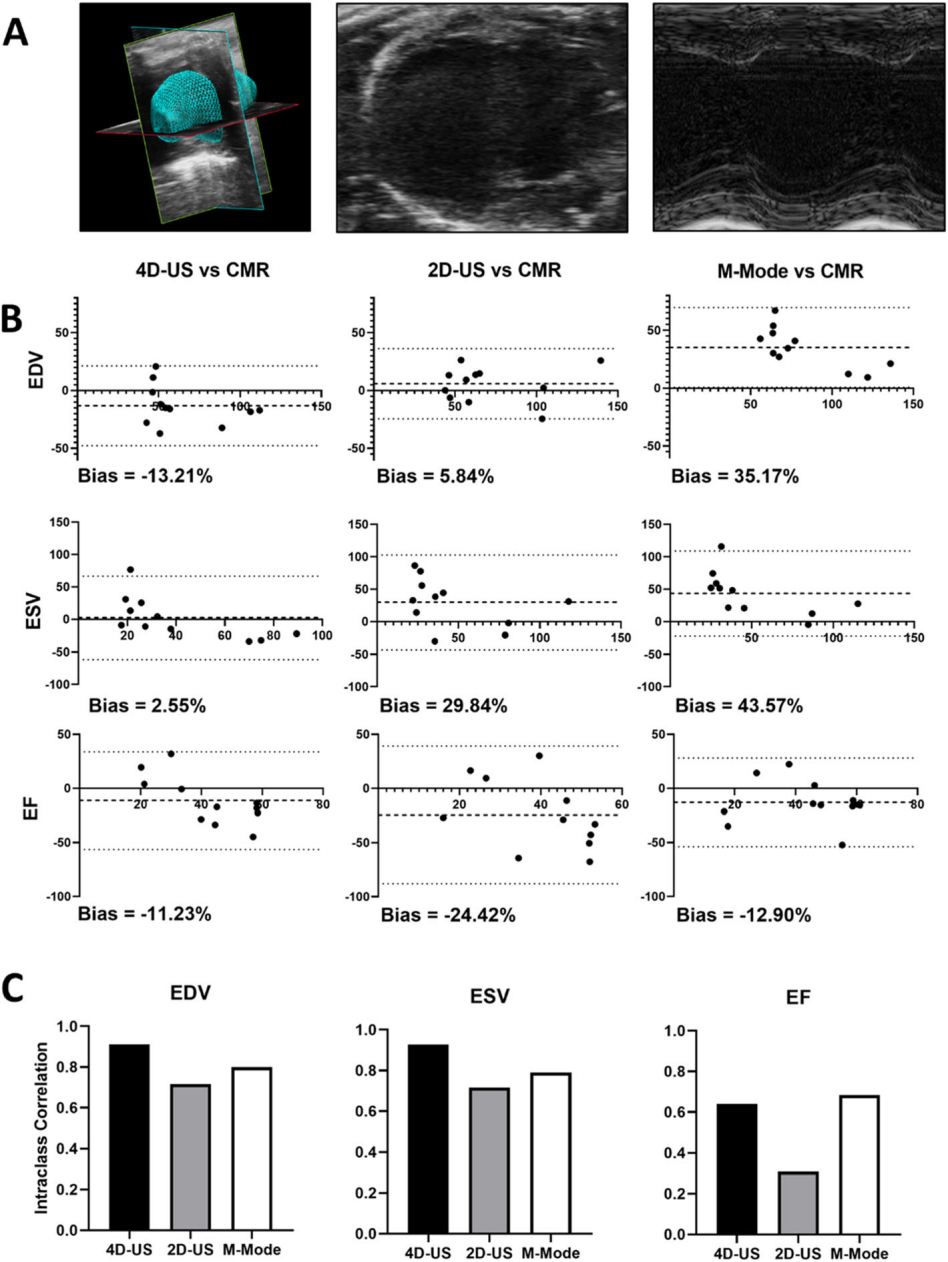
Bland Altman Analysis and Inter-user Variability of ECHO Modalities. **a** Representative ECHO modalities for 4D-US, 2D-US, and M-Mode. **b** Bland-Altman analysis demonstrating the difference between selected measurement modality (4D-US, 2D-US, and M-Mode) and CMR. Data are presented as % bias and dotted lines represent 95% confidence interval. X-axes represent CMR volumetric measurements and Y-axes represent % Difference of ECHO values over CMR values. **c** Inter-user variability between two users for each ECHO modality as calculated by intraclass correlation

To compare imaging modalities following infarction, mice were evaluated by ECHO using traditional M-mode, 2D-US, and 4D-US (representative images in Fig. 1 with mean values reported in Table 2) at baseline, 1 week following CAL, and 4 weeks following CAL. One-day after the 4-week ECHO was performed, a subset of randomly chosen mice underwent CMR to serve as reference standard for volumetric measurements (Fig. 1). Bland-Altman analysis of M-mode, 2D-US, and 4D-US was used to compare the percentage difference of ECHO modalities to CMR (Fig. 2). Of the three ECHO modalities, 4D-US demonstrated the lowest bias when comparing ESV (4D-US Bias: 2.55% LOA: − 61.55 to 66.66, 2D-US Bias: 29.84% LOA: − 43.10 to 102.8, M-Mode Bias: 43.57% LOA: − 21.87 to 109.0; Fig. 2) and EF (4D-US Bias: -11.23% LOA: − 56.26 to 33.80, 2D-US Bias: -24.42% LOA: − 87.99 to 39.16, M-Mode Bias: -12.90% LOA: − 53.80 to 28.00). 4D-US was outperformed by 2D-US when evaluating EDV (4D-US Bias: -13.21% LOA: − 47.71 to 21.28, 2D-US Bias: 5.84% LOA: − 24.47 to 36.15, M-Mode Bias: 35.17% LOA: 0.73 to 69.61). Linear regression was also performed between ECHO modalities and CMR, which demonstrates strong to very strong correlation between 4D-US and CMR (EDV: r = 0.887, ESV: r = 0.943, EF: r = 0.811; Supplemental Fig. 1). 2D-US has lower correlation values for every modality (EDV: r = 0.806, ESV: r = 0.793, EF: r = 0.406). M-Mode correlation is slightly higher than 4D-US for EDV, but lower for ESV and EF (EDV: r = 0.902, ESV: r = 0.882, EF: r = 0.747). All ECHO modalities had similar HRs at the time of image acquisition (4D-US: 459.5 ± 6.3, 2D-US: 459.7 ± 9.2, M-Mode: 451.7 ± 8.3; Table 1) though HR was lower during CMR (356.2 ± 8.2) due to imaging constraints. 4D-US demonstrated the highest ICC for estimation of EDV (4D-US: 0.91, 2D-US: 0.72, M-Mode: 0.80) and ESV (4D-US: 0.93, 2D-US: 0.72, M-Mode: 0.79). M-mode had a higher ICC for evaluation EF (4D-US: 0.64, 2D-US: 0.31, M-Mode: 0.69).

**Table 2:** Mean Volumetric Data at 1 and 4 weeks by Imaging Modality

1 week	MRI	4D-US	2D-US	M-mode
**EF (%)**	–	43.94 ± 2.98	38.83 ± 2.28	48.47 ± 3.26
**SV (**μL**)**	–	16.37 ± 1.38	22.54 ± 1.31	36.54 ± 2.03
**ESV (**μL**)**	–	23.88 ± 3.77	40.12 ± 4.84	42.16 ± 6.97
**EDV (μL)**	–	40.25 ± 4.30	62.66 ± 5.516	94.45 ± 7.43
**4 week**	**MRI**	**4D-US**	**2D-US**	**M-mode**
**EF (%)**	45.89 ± 5.66	38.89 ± 3.41	34.27 ± 3.14	40.04 ± 4.43
**SV (μL)**	27.11 ± 1.46	21.41 ± 1.60	21.76 ± 1.12	33.28 ± 3.21
**ESV (μL)**	41.75 ± 9.57	37.72 ± 6.05	51.44 ± 9.99	57.66 ± 9.45
**EDV (μL)**	68.86 ± 8.96	59.13 ± 6.66	73.20 ± 10.16	94.34 ± 7.82

### Wall motion severity index (WMSI), scar sizing, and strain analysis

**Fig. 3:**
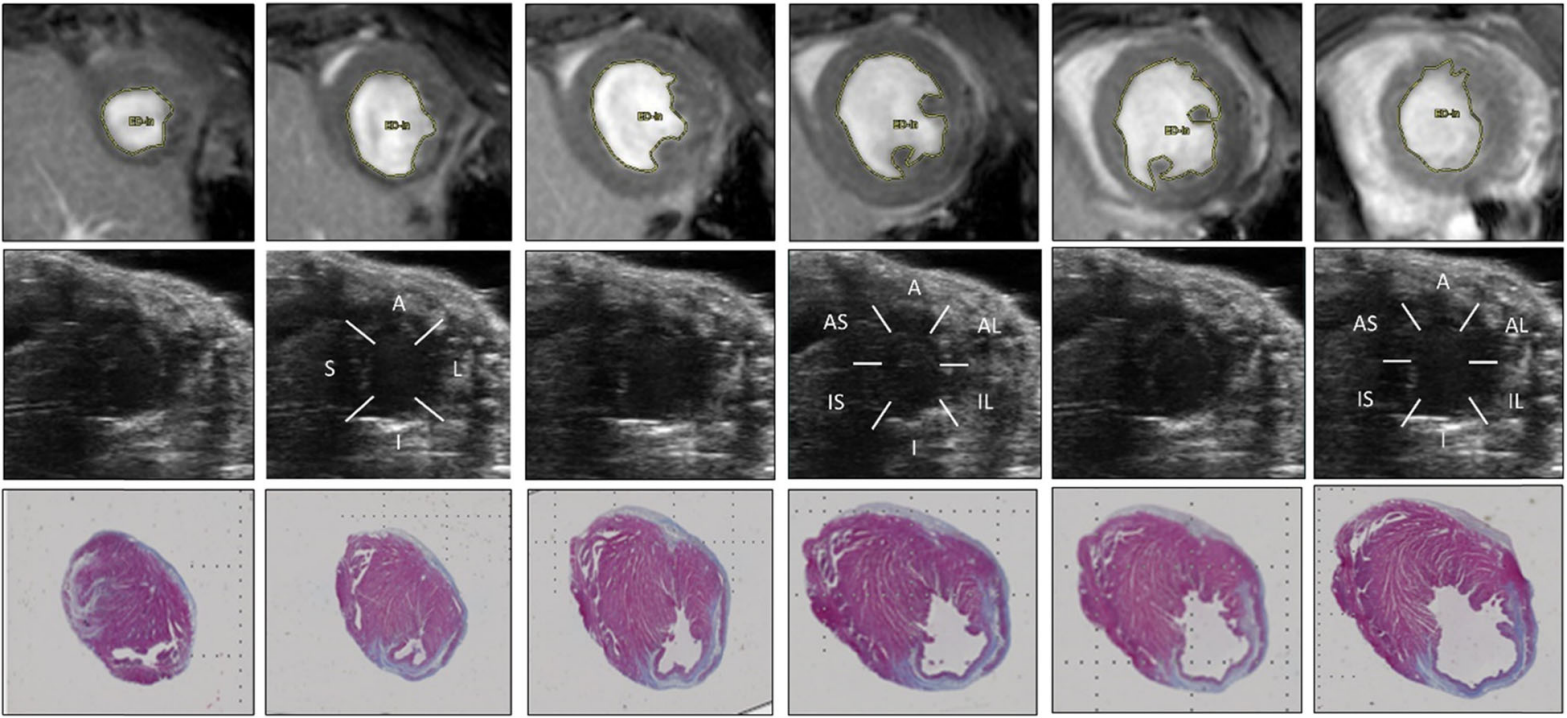
Representative Concentric Images of a Single Heart by CMR, 4D-US, and Histology. CMR (top row), 4D-US (middle row), and trichrome-stained histologic slides taken from a single infarcted heart at ~ 1 mm steps from the apex (left) through basal-ventricle (right). 4D-US images (middle row) are divided into 16 sections for wall-motion score index, labeled as anterior (A), lateral (L), inferior (I), septal (S), anterior-lateral (AL), inferior-lateral (IL), inferior-septal (IS), and anterior-septal (AS)

**Fig. 4:**
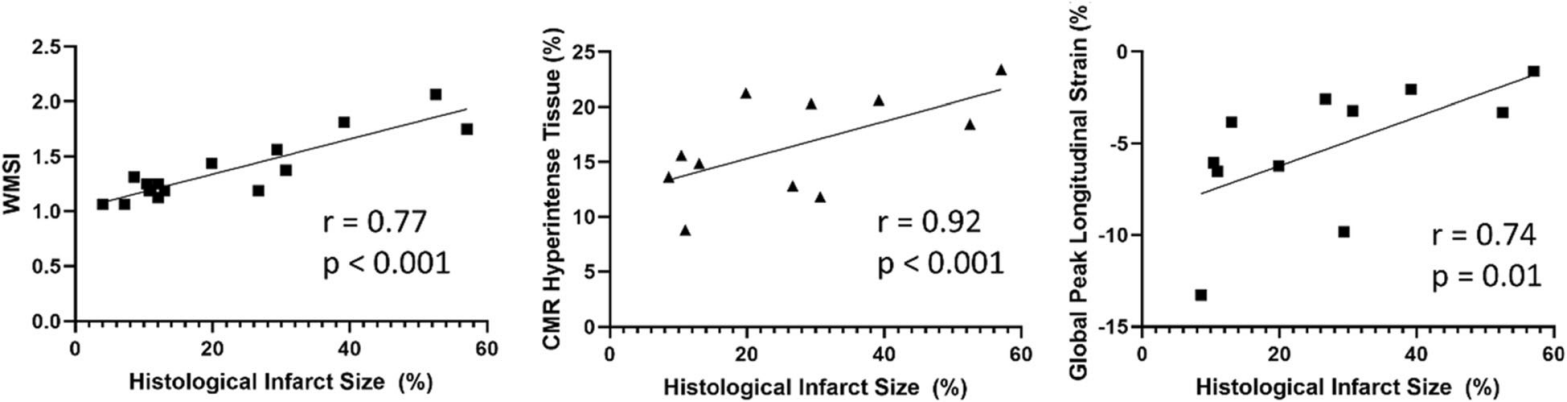
Correlation between WMSI and CMR to Histology. Regression analysis with Spearman rank correlation comparing wall motion score index (WMSI) to histological infarct size (left), CMR hyperintense tissue to histologic infarct size (middle), and global peak longitudinal strain to histologic scar size (right)

To quantify scar size following CAL, we utilized gadolinium-enhanced CMR, step-wise short-axis images obtained via 4D-US, long-axis strain analysis, and trichrome staining of tissue sections, which was used as the gold standard (Fig. 3). Following sacrifice 4 weeks after CAL, a subset of all mice underwent histologic staining to evaluate scar size. Scar size was estimated histologically on trichrome-stained cross sections (Table 1, Fig. 3). The mean scar volume was 21.54 ± 4.07% (Range 4.02–57.00%, Table 1). Late gadolinium-enhanced CMR images yielded a mean volume of 13.53 ± 2.22%% (range 5.52–28.10%, Table 2). WMSI average scores are 1.32 ± 0.05 (range 1.07–2.07, Table 2, *n* = 32). Global longitudinal strain was calculated throughout the myocardium with average strain − 5.27 ± 1.10% (range − 13.29% – -1.072%, Table 2). Spearman rank correlation was used to assess the association of scar size between WMSI, CMR hyperintense regions/LV area, or global peak longitudinal strain to histologic stain assessments (Fig. 4). When compared to histologic scar size, WMSI correlates strongly (r = 0.77, *p* < 0.01), CMR hyperintense volume correlates very strongly (r = 0.90, *p* < 0.001), and longitudinal strain correlates strongly (r = 0.74, *p* = 0.01). Additional strain measurements, including global radial strain, LV dyssynchrony from long-axis imaging, and both circumferential and radial strain from short axis imaging were also calculated and a full comparison of modalities assessed via Spearman correlation (Supplemental Table 2).

## Discussion

Over the past two decades, 3D- and 4D-US have been gaining clinical favor as the technology has advanced and now offers rapid and cost-effective high-resolution imaging [22]. Variations of 3D echocardiography have been shown to have quick and reliable volumetric assessment using semi-automated tracing [23], low inter-user variability [24], and have strong agreement with CMR in normal and abnormal LV’s [25]. Real-time 3D-ultrasound has been shown to outperform 2D-US when compared to single-photon emission computed tomography following myocardial infarction [26] and has allowed for increased detection of LV dyssynchrony [27]. Our work shows that commercially available 4D-US in similarly beneficial in a pre-clinical CAL model, demonstrating higher agreement with CMR and lower inter-user variability than traditional 2D-ECHO modalities.

This study evaluated 4D-US in a mouse model of myocardial infarction 4 weeks after CAL and compared 4D-US endpoints to 2D-US, M-mode, CMR, and histologic parameters. The mouse CAL model presents a challenge for evaluation using traditional echocardiography, given that wall motion abnormalities and dyssynchronous contraction can cause abnormal LV geometry. Traditional M-mode and 2D-US assume ellipsoid geometry for volumetric measurements [6]. Following CAL, mice typically have a hyperdynamic base, static apex, and increased sphericity that limit the effectiveness of the geometric assumptions [17, 28–30]. 4D-US allows for real-time image analysis at multiple planes encompassing the LV that is gated by ECG and respiration [13, 14] to minimize motion artifact during image reconstruction. The result is a data-driven volumetric model that eliminates spatial and temporal assumptions about LV morphology. Our data demonstrate that for calculation of EF and ESV, 4D-US provides overall closer agreement when compared to benchmark CMR than other ECHO modalities 4 weeks after CAL. Additionally, we show that 4D-US has lower inter-user variabilty when measuring ventricular volumes at 4 weeks. Finally, 4D-US allows for simplified evaluation of WMSI, which correlates well with scar size by histologic analysis.

In agreement analyses, we found that 4D-US had lower percentage bias by Bland-Altman analysis than 2D-US and M-mode compared to CMR for the evaluation of ESV and EF at 4 weeks (Fig. 2), suggesting more accurate assessment of volumetric parameters. 4D-US was outperformed by 2D-US in the evaluation of EDV at this time point. However, across the three endpoints evaluated (EDV, ESV, and EF), 4D-US was the most reliable modality in terms of bias and 95% level of agreement. 4D-US has much lower bias than M-mode, which demonstrated > 40% bias in the estimation of ESV. 2D-US bias was improved compared to M-mode, but was characterized by the highest inter-user variability as demonstrated by lowest ICC for the evaluation of EDV, ESV, and EF (Fig. 2c). Inter-user variabilty was lowest using 4D-US for evaluation of EDV and ESV, but was mildly outperformed by M-Mode when evaluating EF (Fig. 2c). In total, our data is consistent with other studies that found that 4D-US provides better accuracy and less inter-user variability when comparing to CMR than 2D-US or M-mode values, making it the prefered echo modality for surgical animal studies.

In considering the limitations of 4D-US, we noted that 4D-US uniformly underestimated EDV and ESV when compared to CMR, though the changes were more pronounced in EDV (Fig. 2b, Table 2). The 4D-US measurements may suffer limitations in surgical mouse models, as the base of the heart is difficult to clearly visualize given the overlying scar following thoracotomy required in the CAL surgery, and may result in lower total volumes. A similar underestimation of volumes was previously noted in evaluation of round “phantoms” using identical software [15], suggesting that a systemic underestimation result of volumes may be present in this modality. Regardless of this underestimation, the modality still represents an direct improvement over other traditional ECHO modes.

Recent work by Russo et al. has shown that automated step-wise short-axis ECHO imaging performed on CAL mice strongly correlates with CMR [17], providing reproducible volumetric evaluations nearly on par with CMR, but at a fraction of the time and cost required for CMR, as well as increased portability and accessibility. Our data support the increased value of this step-wise approach, while incorporating ECG- and respiratory-gating and the higher resolution of the Vevo-3100 for enhanced temporal and spatial resoluation. Soepriatna et al. previously demonstrated similar benefits of gated 4D-US in infarcted mice using manual 3-D reconstruction throughout the cardiac cycle [14]. Previous work by Grune et al. compared wild type mice using 4D-US obtained on Vevo 3100 hardware utilizing custom software analysis to volumetric data from CMR, and found excellent agreement between the modalities [15]. While these studies demonstrated the value of the hardware system and imaging throughout the cardiac cycle, here we use an easily-accessible commercial semi-automated system in a mouse CAL model, which utilizes edge-tracing software to simplify volumetric calculation for users, resulting in both rapid analysis and low inter-user variability.

The 4D-US imaging modality can to quickly and reproducibly quantify scar characteristics using clinical scales of wall-motion, increasing the translational relevance of pre-clinical assessments. Previous works have quantified scar size using ECHO based on wall-motion abnormalities from 2D and 3D-US reconstruction, demonstrating good comparison between ECHO estimations and histologic scar size [31, 32]. We evaluated WMSI using a 16-segment model across three short axis views used in the clinical settings as previously described [20, 21]. We found that WMSI correlates strongly with histologic analysis of scar size (r = 0.77, Fig. 4), whereas longitudinal peak strain also correlates strongly (r = 0.74) and gadolinium-enhanced CMR correlated very strongly (r = 0.90). It should be noted that this pre-clinical model utilizes permanent coronary occlusion. Models of transient occlusion may be complicated by myocardial stunning, which may impair the value of WMSI and its correlation to scar size, and requires further study.

The 4D-US method allows for easy standardization on the WMSI across animals, as the automated step function can be utilized to standardize the height of short-axis imaging. In this study, we used three short-axis images taken 1 mm, 3 mm, and 5 mm from the apex to quantify WMSI. Unfortunately, the 4D-US step-wise images are not compatable with Vevo Strain software, which would allow the user to rapidly obtain staged short-axis strain measurements, which has previously been validated as a predictor of scar size [33]. A full comparison table assessing the correlation between a number of previously validated markers of scar size, including histology, WMSI, CMR, global longitudinal strain, global radial strain, and LV dyssynchrony have been included in Supplemental Table 2.

### Potential caveats

A potential confounder in the study is the use of multiple genotypes on a single background. Specifically, we utilized transgenic mice for the evaluation of volumetric measurements, pooling data from α-MHC-Cre (+) x Flox-TFAM and α-MHC-Cre (−) x Flox-TFAM mice. Because we found no difference between survival, scar size, baseline and 4-week EDV, ESV, or EF between groups (Supplemental Fig. 1, Supplemental Table 1), we included both models for this data set. We believe that the transgenic background does not limit the correlations drawn in this paper between volumetric studies and agrees with the NIH’s effort to reduce animal suffering by utilizing mice readily available in our lab. The CMR and ECHO measurements were completed only on mice following CAL and not on sham animals in this study, and only a limited subset of animals (*n* = 12) underwent all imaging modalities.

It should be noted that there was a HR discrepancy between the ECHO modalities and CMR imaging. While the HR remained consistent between 4D-US, 2D-US, and M-Mode, the HR was nearly 100 bpm slower when undergoing CMR (Table 1) as necessitated by the CMR system. Maintaining physiologic HR is a well known limitation of CMR imaging in the mouse [16]. Sub-physiologic HR’s are associated with elevated ESV, EDV, and decreased EF in healthy mice [34, 35]. However, previous work in a mouse CAL model has shown that EF does not correlate with HR in the CAL mouse in CMR, suggesting that the HR discrepancy may not be as important in the CAL model as it is in in healthy mice [36].

## Conclusions

In this paper we demonstrate that commercially available semi-automated 4D-US provides quick and reliable volumetric measurements of the heart following CAL that compares favorably to CMR values. 4D-US correlates better with CMR than 2D-US and M-Mode for evaluation of volumetric parameters 4 weeks after CAL. 4D-US also allows simple evaluation of WMSI, which is a clinically relevant metric that correlates well with scar size by histologic analysis.

## Supplementary information

**Additional file 1: Figure S1.** Linear Regression Analysis of 4-Week ECHO Modalities Compared to CMR. Linear regression with correlation coefficients evaluating volumetric measurements of 4D-US, 2D-US, and M-Mode at 4 weeks to CMR at 4 weeks.

**Additional file 2: Figure S2**. Transgenic Mouse Lines have No Differences in EDV, ESV, or EF. α-MHC-Cre (+) x Flox-TFAM and α-MHC-Cre (−) x Flox-TFAM mice were compared at baseline and Week 4 following CAL by 4D-US, 2D-US, and M-mode and demonstrate no significant changes by any modality when comparing EDV, ESV, and EF.

**Additional file 3: Table S1.** Comparison of ESV, EDV, EF, Scar Size, and survival between transgenic groups.

**Additional file 4: Table S2.** Spearman Correlation Values and *p*-Values Between Modalities for Assessing Scar Size. Histologic Sections, WMSI from 4D-US, Hyperintense Tissue on CMR, Longitudinal Strain, Long Axis Radial Strain, Long-Axis LV Dyssynchrony, Short Axis Radial Strain, and Short Axis Circumferential Strain are compared to each other modality

## Data Availability

The datasets used and analyzed during the current study are available from the corresponding author on reasonable request.

## Abbreviations

2D-US: 2-Dimensional Ultrasound
3D: 3-Dimensional
4D-US: 4-Dimensional Ultrasound
A: Anterior
AL: Anterior Lateral
AS: Anterior Septal
CAL: Coronary Artery Ligation
CMR: Cardiac Magnetic Resonance
CO: Cardiac Output
Echocardiography: ECHO
ED: End Diastole
EDV: End Diastolic Volume
EF: Ejection Fraction
ES: End Systole
ESV: End Systolic Volume
FA: Flip Angle
FOV: Field of View
H&E: Hematoxylin and Eosin
I: Inferior
ICC: Intraclass Correlation
IL: Inferior Lateral
IS: Inferior Septal
L: Lateral
Level of agreement: LOA
LV: Left Ventricle
LVV: Left Ventricular blood volume
MI: Myocardial Infarction
M-Mode: Motion-mode
S: Septal
SV: Stroke Volume
TE: Echo Time
TR: Repetition Time
WMSI: Wall Motion Severity Index
