# Supplementary material for: Commercial 4-dimensional echocardiography for murine heart volumetric evaluation after myocardial infarction
Source: Cardiovasc Ultrasound. 2020 Mar 12;18:9. doi: 10.1186/s12947-020-00191-5 (PMC7068892; doi:10.1186/s12947-020-00191-5)
Supplement: Supplementary file 1 — Additional file 1: Figure S1. Linear Regression Analysis of 4-Week ECHO Modalities Compared to CMR. Linear regression with correlation coefficients evaluating volumetric measurements of 4D-US, 2D-US, and M-Mode at 4 weeks to CMR at 4 weeks. [file 12947_2020_191_MOESM1_ESM.docx]

**
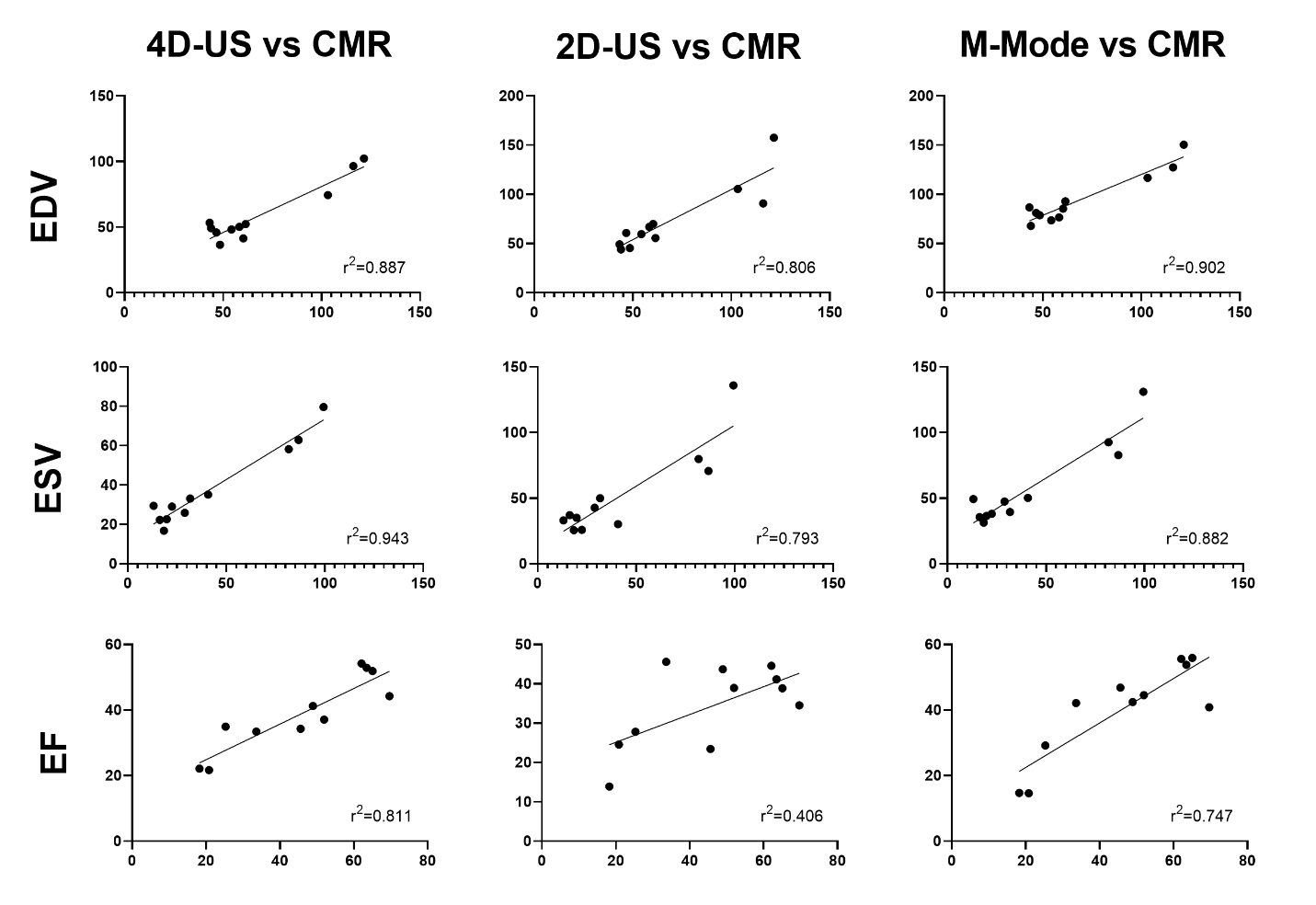
**

**Supplemental Figure 1. Linear Regression Analysis of 4-Week ECHO Modalities Compared to CMR**. Linear regression with correlation coefficients evaluating volumetric measurements of 4D-US, 2D-US, and M-Mode at 4 weeks to CMR at 4 weeks.
