# Supplementary material for: Commercial 4-dimensional echocardiography for murine heart volumetric evaluation after myocardial infarction
Source: Cardiovasc Ultrasound. 2020 Mar 12;18:9. doi: 10.1186/s12947-020-00191-5 (PMC7068892; doi:10.1186/s12947-020-00191-5)
Supplement: Supplementary file 2 — Additional file 2: Figure S2. Transgenic Mouse Lines have No Differences in EDV, ESV, or EF. α-MHC-Cre (+) x Flox-TFAM and α-MHC-Cre (−) x Flox-TFAM mice were compared at baseline and Week 4 following CAL by 4D-US, 2D-US, and M-mode and demonstrate no significant changes by any modality when comparing EDV, ESV, and EF. [file 12947_2020_191_MOESM2_ESM.docx]

**
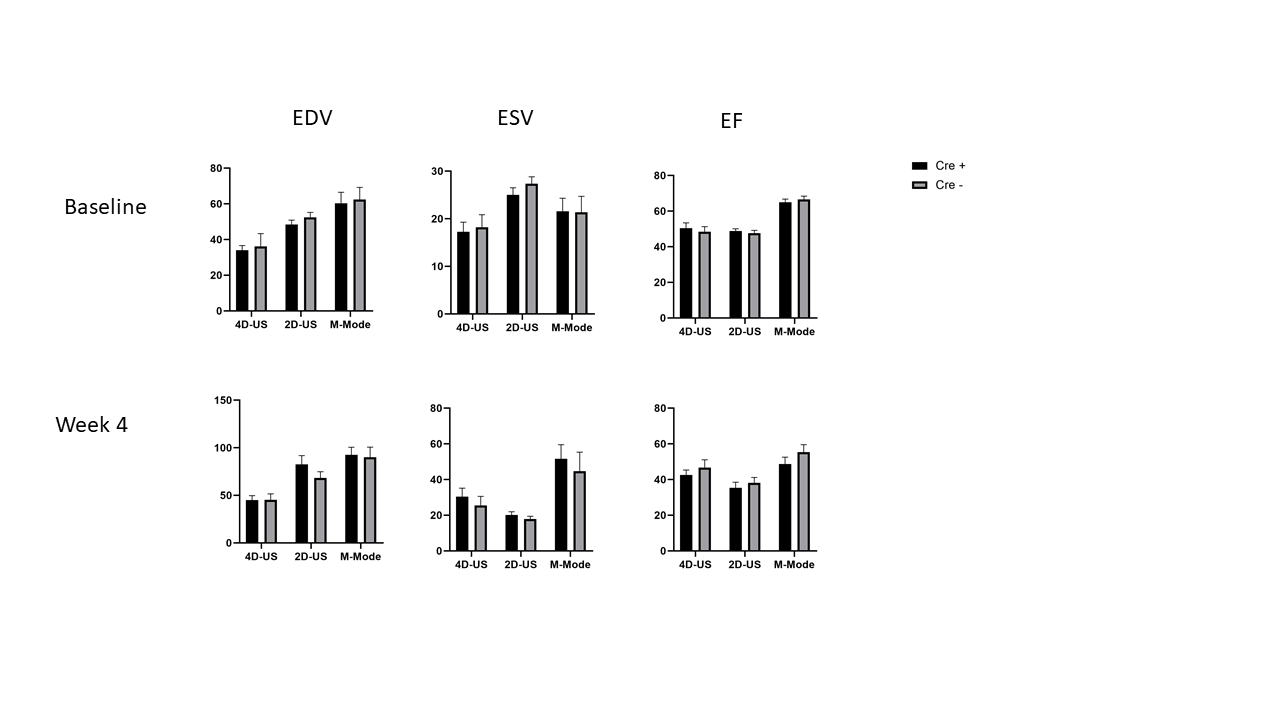
**

**Supplemental Figure 2. Transgenic Mouse Lines have No Differences in EDV, ESV, or EF**. α-MHC-Cre (+) x Flox-TFAM and α-MHC-Cre (-) x Flox-TFAM mice were compared at baseline and Week 4 following CAL by 4D-US, 2D-US, and M-mode and demonstrate no significant changes by any modality when comparing EDV, ESV, and EF.
