# Supplementary material for: Commercial 4-dimensional echocardiography for murine heart volumetric evaluation after myocardial infarction
Source: Cardiovasc Ultrasound. 2020 Mar 12;18:9. doi: 10.1186/s12947-020-00191-5 (PMC7068892; doi:10.1186/s12947-020-00191-5)
Supplement: Supplementary file 3 — Additional file 3: Table S1. Comparison of ESV, EDV, EF, Scar Size, and survival between transgenic groups. [file 12947_2020_191_MOESM3_ESM.docx]

**Supplemental Table 1. Comparison of ESV, EDV, EF, Scar Size, and survival between transgenic groups**

|  |  | **MHC (+) x Flox-TFAM** | **MHC (-) x Flox-TFAM** | **p-value** |
| --- | --- | --- | --- | --- |
| **Survival** |  | 86.3% | 88.2% |  |
| **Baseline Wt (g)** |  | 24.91 ± 0.84 | 25.79 ± 0.728 | 0.46 |
| **4 Week Wt (g)** |  | 25.11 ± 0.83 | 26.03 ± 0.68 | 0.46 |
| **Scar Size (%)** |  | 16.11 ± 6.93 | 25.77 ± 4.73 | 0.27 |
| **WMSI** |  | 1.36 ± 0.07 | 1.27 ± 0.08 | 0.39 |
| **Baseline 2D-US** | EDV (µL) | 48.41 ± 2.50 | 52.52 ± 2.71 | 0.28 |
|  | ESV (µL) | 25.00 ± 1.52 | 27.36 ± 1.47 | 0.28 |
|  | EF (%) | 48.81 ± 1.23 | 47.73 ± 1.47 | 0.58 |
| **4 Week 4D-US** | EDV (µL) | 44.95 ± 4.79 | 45.27 ± 6.29 | 0.96 |
|  | ESV (µL) | 30.45 ± 4.73 | 27.19 ± 6.09 | 0.65 |
|  | EF (%) | 42.58 ± 2.81 | 46.68 ± 4.43 | 0.42 |
