# Supplementary material for: Commercial 4-dimensional echocardiography for murine heart volumetric evaluation after myocardial infarction
Source: Cardiovasc Ultrasound. 2020 Mar 12;18:9. doi: 10.1186/s12947-020-00191-5 (PMC7068892; doi:10.1186/s12947-020-00191-5)
Supplement: Supplementary file 4 — Additional file 4: Table S2. Spearman Correlation Values and p-Values Between Modalities for Assessing Scar Size. Histologic Sections, WMSI from 4D-US, Hyperintense Tissue on CMR, Longitudinal Strain, Long Axis Radial Strain, Long-Axis LV Dyssynchrony, Short Axis Radial Strain, and Short Axis Circumferential Strain are compared to each other modality [file 12947_2020_191_MOESM4_ESM.docx]

**Supplemental Table 2. Spearman Correlation Values and p-Values Between Modalities for Assessing Scar Size.** Histologic Sections, WMSI from 4D-US, Hyperintense Tissue on CMR, Longitudinal Strain, Long Axis Radial Strain, Long-Axis LV Dyssynchrony, Short Axis Radial Strain, and Short Axis Circumferential Strain are compared to each other modality

|  | **Histology** | **WMSI** | **CMR** | **Longitudinal Strain** | **Long Axis Radial Strain** | **LV Dyssynchrony** | **Short Axis Radial Strain** |
| --- | --- | --- | --- | --- | --- | --- | --- |
| **Histology** | - | - | - | - | - | - | - |
| **WMSI** | r=0.77  p<0.001 | - | - | - | - | - | - |
| **CMR** | r=0.90  p<0.001 | r=0.83  p=0.003 | - | - | - | - | - |
| **Longitudinal Strain** | r=0.74  p=0.013 | r=0.30  p=0.36 | r=0.61  p=0.052 | - | - | - | - |
| **Radial Strain** | r=0.56  p=0.081 | r=0.17  p=0.61 | r=0.44  p=0.173 | r=0.92  p<0.001 | - | - | - |
| **LV Dyssynchrony** | r=0.03  P=0.956 | r=0.61  p=0.049 | r=0.68  p=0.025 | r=0.43  p=0.18 | r=0.76  p=0.009 | - | - |
| **Short Axis Radial Strain** | r=0.68  p =0.025 | r=0.43  p=0.188 | r=0.79  p=0.006 | r=0.75  p=0.010 | r=0.70  p=0.020 | r=0.75  p=0.010 | - |
| **Short Axis Circumferential Strain** | r=0.66  p=0.031 | r=0.53  p=0.097 | r=0.61  p=0.052 | r=0.59  p=0.061 | r=0.52  p=0.107 | r=0.54  p=0.09 | r=0.69  p=0.022 |
